# Hybrid feature selection framework for enhanced credit card fraud detection using machine learning models

**DOI:** 10.1371/journal.pone.0326975

**Published:** 2025-07-16

**Authors:** Al Mahmud Siam, Pankaj Bhowmik, Md Palash Uddin

**Affiliations:** Department of Computer Science and Engineering, Hajee Mohammad Danesh Science and Technology University, Dinajpur, Bangladesh; Majmaah University, SAUDI ARABIA

## Abstract

Electronic payment methods are increasingly prevalent worldwide, facilitating both in-person and online transactions. As credit card usage for online payments grows, fraud and payment defaults have also risen, resulting in significant financial losses. Detecting fraudulent transactions is challenging due to the highly imbalanced nature of transaction datasets, where fraudulent activities constitute only a small fraction of the data. To address this, we propose a novel hybrid feature selection framework designed to enhance the performance of machine learning models in credit card fraud detection. Our framework integrates three complementary feature selection techniques: Pearson correlation, information gain (IG), and random forest importance (RFI), each optimized for the dataset‘s characteristics. Pearson Correlation eliminates redundancy by removing highly correlated features, while IG and RFI evaluate the relevance of the remaining features. A union operation combines the most informative features from these methods, ensuring comprehensive and efficient feature selection. To validate the proposed approach, we test it on five diverse datasets with varying characteristics and imbalance levels, employing five state-of-the-art machine learning algorithms: Random Forest (RF), Extra Trees (ET), XGBoost (XGBC), AdaBoost, and CatBoost. We primarily propose this work for PCA-transformed datasets, but for the validation of our research, we also apply it to a real-world dataset. The results demonstrate that our methodology outperforms existing baseline approaches, achieving superior fraud detection performance across all datasets. Our findings highlight the robustness and adaptability of the proposed framework, offering a practical solution for real-world fraud detection systems. Additionally, we believe that our proposed framework can serve as a decision support system for the detection of fraudulent transactions in real-time credit cards, with the potential to make a substantial contribution to the business industry.

## 1 Introduction

In the modern era, technology impacts nearly every aspect of our lives, including education, healthcare, economics, finance, trade, industry, politics, and entertainment. Consumers’ methods to perform transactions have undergone significant changes and growth in recent years. The evolution of modern lifestyles, technological advancements, and the widespread adoption of online applications have all contributed to the rise of electronic commerce (e-commerce) and online credit card transactions for purchases and payments [1, 2]. The trend towards a cashless society is undeniable, with future transactions increasingly shifting away from traditional cash-based methods. Consequently, customers will no longer need to carry cash for in-store purchases, compelling businesses to enhance their infrastructure to accommodate diverse payment processing methods. This shift is expected to intensify in the coming years [3].

However, in addition to these advancements, there has been a notable increase in security issues associated with credit card transactions. Credit card fraud—a form of identity theft where unauthorized individuals exploit a credit card or its account information to complete transactions—has become a major concern for both financial institutions and their customers [3]. Fraudulent transactions occur worldwide and have resulted in substantial financial losses. Alarmingly, in 2020, global losses from such transactions amounted to 28.58 billion USD, with the United States alone reporting losses of 11 billion USD. From 2011 to 2021, global losses surged from 9.84 billion USD to 32.39 billion USD, and they are projected to reach a cumulative total of 408.50 billion USD over the next decade. In Malaysia, fraudulent credit card transactions cost the banking industry 51.3 million RM in 2016, and 12.8% of credit cardholders reportedly struggled to meet minimum balance payments [4].

To mitigate these challenges, financial institutions and credit card users require robust fraud detection systems capable of preventing fraudulent transactions. Automated anomaly detection systems, leveraging machine learning (ML) algorithms, play a critical role in addressing this issue [5–7]. ML, a subfield of artificial intelligence, uses computational methods to identify patterns in historical data and make predictions [8]. ML algorithms are generally categorized as supervised, unsupervised, or reinforcement learning, with supervised algorithms being the most widely applied in credit card fraud detection [9,10]. Detecting fraudulent transactions is particularly challenging due to their evolving nature and the inherent imbalance in datasets, where fraudulent transactions represent a small fraction of total transactions [3].

A variety of ML models have been employed for fraud detection, including Decision Trees (DT), Random Forest (RF), ANN, Naive Bayes (NB), CatBoost (CB), Adaptive Boosting (AdaBoost), Extreme Gradient Boosting (XGBC), Logistic Regression (LR) and Support Vector Machines (SVM). In addition, recent advances in deep learning (DL), a subset of ML, have introduced neural network-based models that mimic human cognitive processes. Popular DL algorithms include Convolutional Neural Networks (CNN), Multilayer Perceptrons (MLP), Recurrent Neural Networks (RNN), Long-Short-Term Memory (LSTM) and Gated Recurrent Units (GRU) [11]. In this paper, we utilize five supervised ML algorithms—RF, Extra Trees (ET), XGBC, AdaBoost, and CatBoost—along with an ensemble technique that combines these models to detect credit card fraud. We develop a hybrid feature selection technique that not only enhances model performance but also effectively addresses data imbalance issues. To validate our methodology, we experiment with five diverse datasets, varying in size (large, medium, and small) and imbalance ratios (0.172%, 14.56%, 30%, 50%, and 55.51%). This ensures the robustness of our proposed sampling technique. Our contributions are as follows:

- We review the limitations of existing research works on credit card fraud detection, with a focus on ML and DL techniques.
- We propose a manually designed hybrid feature selection technique to retain only the most relevant features.
- By reducing the number of features, we achieve faster training times and minimize computational complexity.
- We develop an empirical framework that could benefit banks, merchants, cardholders, insurance companies, and other stakeholders.
- Our proposed method is tested on five datasets, demonstrating superior performance compared to existing approaches.

The remainder of this paper is organized as follows: Sect 2 discusses related studies and their limitations. Sect 3 presents the datasets used in this study. Sect 4 describes our proposed methodology, and Sect 5 outlines the performance metrics. Sect 6 discusses the simulation results and comparisons with existing methods. Finally, Sect 7 concludes the study and outlines directions for future research.

## 2 Related work

Recent years have witnessed a surge in research exploring the application of ML and DL techniques to detect fraudulent transactions. Various approaches have been proposed, particularly in credit scoring, to establish effective fraud detection methodologies. This section provides an overview of key studies, focusing on their ML models and approaches to credit card fraud detection.

Ileberi *et al*. [7] introduced a genetic algorithm (GA) for feature selection, followed by applying five ML models—DT, RF, LR, ANN, and NB—to detect credit card fraud. In [23], the authors described the development and deployment of a fraud detection system integrating manual and automatic classification, comparing multiple ML methods. Makki *et al*. [24] conducted an extensive experimental study addressing the imbalance classification problem. Xu *et al*. [25] proposed an ensemble strategy to balance training data for extreme learning machine (ELM) classifiers, employing a weighting method based on generalized fuzzy soft sets (GFSS) theory. In [13], the authors utilized Isolation Forest (IF) and Local Outlier Factor (LOF) algorithms to identify fraudulent transactions. In this study [26], the author introduced a method that combines ensemble learning with a generative adversarial network (GAN), enhanced by Ensemble Synthesized Minority Oversampling Techniques (ESMOTE-GAN).

In [14], researchers demonstrated that combining the Classification and Regression Trees (CART) algorithm with Particle Swarm Optimization (PSO) achieved higher accuracy than CART combined with NB. Baabdullah *et al*. [1] developed ML and DL algorithms, including RF, CNN, and LSTM, optimized with ADAM, SGD, and MSGD. Their CNN-SGD combination achieved 97% accuracy. Sohony *et al*. [65] designed an ensemble majority voting technique using three feedforward neural networks and two RF classifiers. Randhawa *et al*. [69] introduced hybrid methods combining ensemble majority voting with AdaBoost. Table 1 summarizes key studies, highlighting the ML models used, datasets, and their identified limitations.

**Table 1:** Recent benchmark studies.

Ref.	Dataset	Model	Performance	Limitation
[7]	European cardholders 2013 (ECC-2013)	GA-RF	F1-score (F) = 82.41%, AUC = 95%	GA is a time-consuming process for large datasets like credit cards, computation complexity for selection, crossover, and mutation.
[12]	IEEE-CIS	Adaboost + LGBM	AUC = 0.82, F = 0.77, Recall (R) = 0.64	Need better performance. Both AdaBoost and LGBM required multiple hyperparameter tunes that increased model complexity.
[13]	German Dataset	LOF	ACC = 70.60%, F = 41%, R = 50%	Low performance.
[14]	European cardholders 2023 (ECC-2023)	CART+ PSO	ACC = 99.97%, AUC = 100%, R = 100%	The cross-validation metric is not implemented, and the performance is not good enough.
[15]	ECC-2013	KNN	ACC = 99.82%, R = 03.93%, Precision (P) = 71.42%	Didn’t handle the imbalance dataset. Very low recall score, imbalance dataset, not only depend on accuracy score, time-consuming.
[16]	ECC-2013	KNN	ACC = 75.1%, F = 44.1%	Very low accuracy score, inappropriate for fraud detection using KNN.
[17]	ECC-2013	DT	AUC = 90%	Only one performance metric used here.
[18]	Australian dataset	XGBoost-tree-structured Parzen estimator (TPE)	ACC = 87.92%, AUC = 65.71%	Low AUC score, Bayesian hyper-parameter optimization has high computational costs.
[18]	German dataset	XGBoost - TPE	ACC = 77.34%, AUC = 30.62%	Low accuracy score and AUC score.
[19]	ECC-2013	KNN	Sensitivity = 81.19%, P = 91.11%	Imbalance dataset, KNN is time-consuming.
[20]	ECC-2013	CS-SVM	ACC = 98%, R = 99%, F = 93%, P = 98%	High computation cost and high computation cost for large datasets.
[21]	Nigerian bank	LR	AUC = 75%	Not enough performance metrics; only the AUC score cannot evaluate a model.
[22]	Taiwan dataset	RUSMRN	ACC = 79.73%, Sensitivity = 53.36%	Lack of performance metrics, low accuracy score, model complexity.

Most of the papers mentioned above have limitations, as identified through our review and analysis. Some do not address the issue of imbalanced datasets, while others rely solely on a single performance metric for evaluation. In addition, certain studies do not employ feature selection methods, and some proposed models suffer from high computational time and complexity. To overcome these challenges, we propose a novel hybrid feature selection methodology designed to address all the aforementioned issues. In this study, we utilize five datasets, each with distinct characteristics: one is highly imbalanced, one is fully balanced, one is nearly balanced, and the remaining two have moderate imbalance levels. Our manually designed hybrid feature selection methodology effectively reduces computation time and complexity while maintaining robust performance. Unlike previous studies, we do not rely on a single performance metric to evaluate machine learning models. Instead, we use six different performance metrics to provide a comprehensive evaluation, ensuring a more accurate and reliable model selection process.

## 3 Datasets

**Fig 1:**
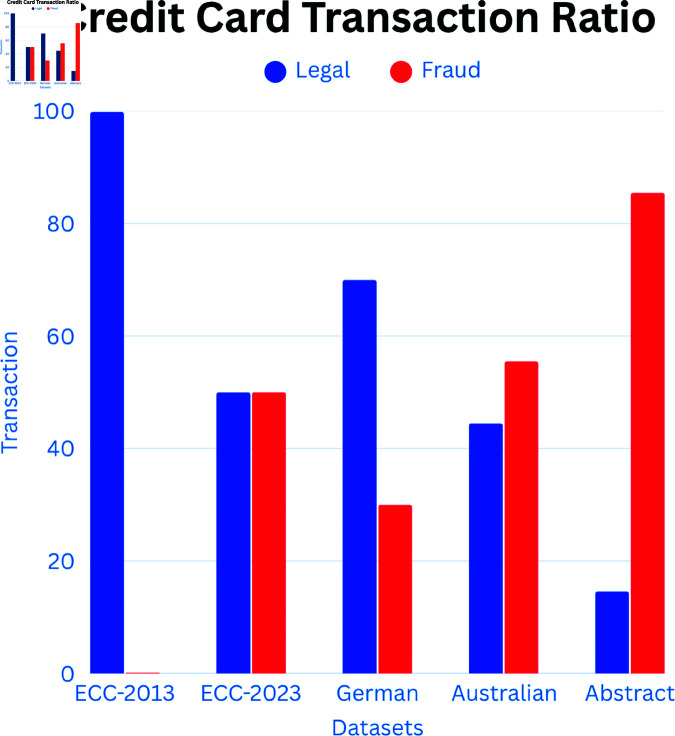
Visualization of different datasets.

We have validated our proposed methodology using multiple datasets. In this paper, we utilized five datasets that are widely referenced in existing studies. Fig 1 visually represents all these datasets. Table 2 lists all the features used across these datasets, and Table 3 summarizes their key characteristics. The details of each dataset are as follows:

**Table 2:** Dataset features.

Dataset Name	Features
European Cardholders 2013	‘Time’, ‘V1’, ‘V2’, ‘V3’, ‘V4’, ‘V5’, ‘V6’, ‘V7’, ‘V8’, ‘V9’, ‘V10’, ‘V11’, ‘V12’, ‘V13’, ‘V14’, ‘V15’, ‘V16’, ‘V17’, ‘V18’, ‘V19’, ‘V20’, ‘V21’, ‘V22’, ‘V23’, ‘V24’, ‘V25’, ‘V26’, ‘V27’, ‘V28’, ‘Amount’, ‘Class’
European Cardholders 2023	‘Id’, ‘V1’, ‘V2’, ‘V3’, ‘V4’, ‘V5’, ‘V6’, ‘V7’, ‘V8’, ‘V9’, ‘V10’, ‘V11’, ‘V12’, ‘V13’, ‘V14’, ‘V15’, ‘V16’, ‘V17’, ‘V18’, ‘V19’, ‘V20’, ‘V21’, ‘V22’, ‘V23’, ‘V24’, ‘V25’, ‘V26’, ‘V27’, ‘V28’, ‘Amount’, ‘Class’
German Dataset	‘existingchecking’, ‘duration’, ‘credithistory’, ‘purpose’, ‘creditamount’, ‘savings’, ‘employmentsince’, ‘installmentrate’, ‘statussex’, ‘otherdebtors’, ‘residencesince’, ‘property’, ‘age’, ‘otherinstallmentplans’, ‘housing’, ‘existingcredits’, ‘job’, ‘peopleliable’, ‘telephone’, ‘foreignworker’, ‘classification’
Australian Dataset	‘A1’, ‘A2’, ‘A3’, ‘A4’, ‘A5’, ‘A6’, ‘A7’, ‘A8’, ‘A9’, ‘A10’, ‘A11’, ‘A12’, ‘A13’, ‘A14’, ‘Class’
Abstract Dataset	‘Average Amount/transaction/day’, ‘Transaction_amount’, ‘Is declined’, ‘Total Number of declines/day’, ‘isForeignTransaction’, ‘isHighRiskCountry’, ‘Daily_chargeback_avg_amt’, ‘6_month_avg_chbk_amt’, ‘6-month_chbk_freq’, ‘isFraudulent’

**Table 3:** Characteristics of different datasets.

Dataset Name	Total Samples	Genuine Transactions	Fraud Transactions	Ratio of Fraudulent Transactions	Total No. of Features
European Cardholders 2013	284,807	284,315	492	0.172%	30
European Cardholders 2023	568,630	284,315	284,315	50%	31
German Dataset	1,000	700	300	30%	21
Australian Dataset	690	307	383	55.51%	15
Abstract Dataset	3,075	448	2,627	85.43%	12

**European Cardholders 2013.** This dataset [27] contains a total of 284,807 samples, of which 492 were identified as fraudulent by European cardholders in September 2013. It exhibits a significant imbalance, with fraudulent transactions accounting for only 0.172% of all transactions. Table 2 lists all features of this dataset. Except for ‘Time’ and ‘Amount’, all features undergo PCA transformation to maintain the privacy of customer information and transactional details. The Class feature indicates the type of transaction, where a value of 0 denotes a legal transaction and 1 represents a fraudulent transaction. In this paper, we refer to this dataset as the European Cardholders 2013 dataset.

**European Cardholders 2023.** This dataset [28] comprises 568,630 rows and 31 columns. It is fully balanced, eliminating the need for the SMOTE technique. Except for ‘Time’, ‘Amount’, and ‘id’, all features undergo PCA transformation to ensure the privacy of sensitive data. The Class feature is the dependent variable, with 1 indicating fraudulent transactions and 0 denoting legal transactions. The ‘id’ column is removed as it has no impact on the analysis. In this study, we refer to this dataset as the European Cardholders 2023 dataset.

**German Dataset.** The third dataset used in this study is the German credit card dataset, a real-world dataset [29], which consists of 21 features, 13 of which are categorical and 8 are numerical [13]. We applied one-hot encoding to convert categorical features into numerical ones, resulting in 62 features, including the classification feature. This dataset contains 1,000 credit card transactions, with 700 classified as normal and 300 as fraudulent, representing 30% fraudulent transactions. In our research, we refer to this dataset as the German Dataset. This dataset offers a valuable benchmark as it reflects real-world transaction data without any dimensionality reduction, pre-processing transformations, or modifications, unlike many synthetic datasets or PCA-transformed datasets commonly used in the literature.

**Australian Dataset.** The fourth dataset [30] consists of 15 features and 690 instances, including 307 legal and 383 fraudulent transactions. All attribute names and values have been changed to meaningless symbols to protect the confidentiality of the data. The dataset is not significantly imbalanced, with fraudulent transactions comprising 55.51% of the data, thus eliminating the need for the SMOTE technique. To ensure confidentiality, feature names and values in this dataset have been replaced with non-meaningful symbols [31]. In this study, we refer to this dataset as the Australian Dataset.

**Abstract Dataset.** The final dataset [32] contains 3,075 samples and 12 features. Of these, 448 samples are fraud cases, accounting for 14.6% of the total. The dataset includes the Merchant_id features, which are irrelevant for fraud detection, and Transaction_date, which contains NaN values. These two features were removed, leaving 10 features, including four categorical variables with values ‘Y’ and ‘N’. We replaced ‘Y’ with 1 and ‘N’ with 0 during preprocessing. This dataset, presented by the author [33], is both abstract and relatively small. In this paper, we refer to it as the Abstract Dataset.

Among the five datasets, the European Cardholders 2013 and 2023 datasets apply PCA to all features (except ‘Time’ and ‘Amount’) to anonymize sensitive transaction details. PCA transforms original variables into uncorrelated components, making it infeasible to reverse-engineer or identify individual user behavior. This obfuscation ensures compliance with privacy standards by structurally removing direct personal identifiers.

## 4 Proposed methodology

**Fig 2:**
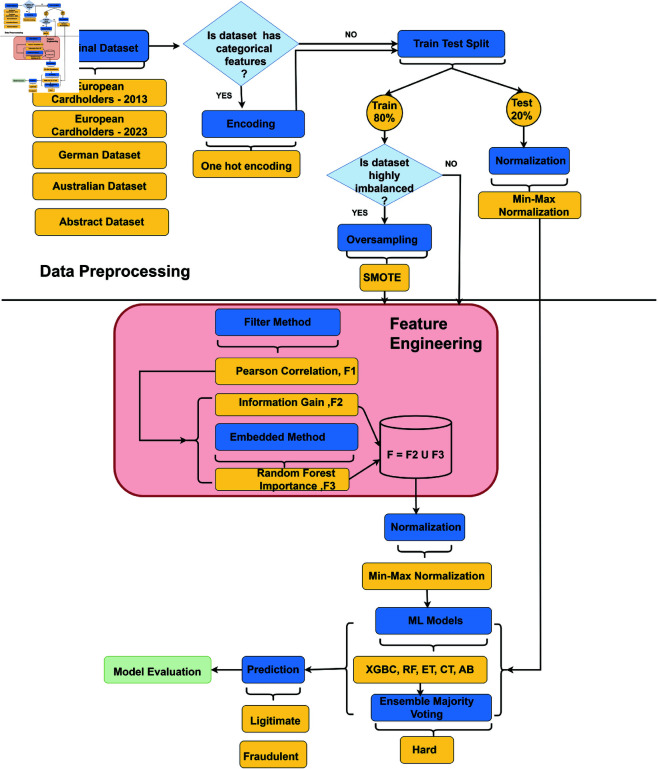
Architecture of the proposed methodology.

**Fig 3:**
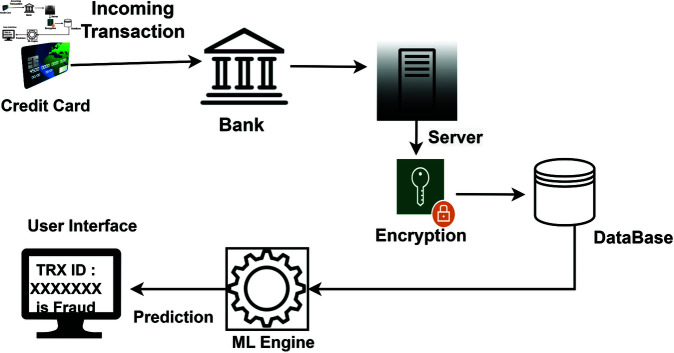
Overview of the proposed system diagram.

This section outlines the proposed framework of our methodology, which consists of two primary components: data pre-processing and feature engineering. Subsequently, a machine learning system is implemented to classify transactions. Fig 2 illustrates the workings of this approach, while Fig 3 provides an overview of the entire fraud detection system’s flow. Note that no human or animal subject is related to this research.

### 4.1 Data preprocessing

Data preprocessing encompasses various techniques applied to raw data to prepare it for further analysis [34]. Raw data often contains imperfections, incompleteness, and inconsistencies that can hinder accurate analysis. By employing data preprocessing strategies, the quality of the data is enhanced, improving the accuracy and efficiency of subsequent mining processes [35]. As a critical step in data mining, data preprocessing involves preparing and transforming data into a suitable format for analysis. This process aims to reduce data volume, identify relationships within the data, normalize values, remove outliers, and extract relevant features. It includes strategies such as data cleaning, integration, transformation, and reduction [36]. In our work, we applied data preprocessing techniques as necessary. Specifically, we utilized oversampling, normalization, and encoder methods to prepare the datasets for analysis in this study.

**Algorithm 1. Data preprocessing with encoding and SMOTE.**

**Input:** Dataset(*O*, *F*)    ⊳ *O* := Observations, *F* := Features

**Output:** $Tx_{\text{SMOTE}}$, $Ty_{\text{SMOTE}}$

1: D←Dataset(O,F)

2: Y←D[Target]    ⊳ Target variable

3: X←D⧵{Y}    ⊳ Features (independent variables)

4: **if** X has Categorical Features **then**    ⊳ Encoding categorical variables

5:   *Encoder* ← **OneHotEncoder**(handle_unknown=‘ignore’)

6:   X←Encoder .fit_transform}(X)    ⊳ Apply one-hot encoding to features

7: **end if**

8: $\left(T_{x},T_{y}),(T_{x}^{\prime },T_{y}^{\prime })\leftarrow Train\_Test\_Split(X_{\text{encoded}},Y\right)$    ⊳ Split dataset into training and testing sets

9: **if is_imbalanced**(D) **then**    ⊳ Check if the dataset is imbalanced

10:   $\left(Tx_{\text{SMOTE}},Ty_{\text{SMOTE}})\leftarrow Apply\_SMOTE(T_{x},T_{y}\right)$    ⊳ SMOTE applied to the training set to handle imbalance

11: **else**

12:   $\left(Tx_{\text{SMOTE}},Ty_{\text{SMOTE}})\leftarrow (T_{x},T_{y}\right)$    ⊳ No need for SMOTE if the dataset is balanced

13: **end if**

14: **Retrun** $\left(Tx_{\text{SMOTE}},Ty_{\text{SMOTE}}\right)$

#### 4.1.1 Oversampling.

In classification problems, the issue of imbalanced datasets arises when the number of instances representing different classes varies significantly [37]. Most learning algorithms are designed to achieve high predictive accuracy and strong generalization capabilities. However, this inductive bias presents a considerable challenge in the context of imbalanced data, as models tend to favor the majority class, leading to poor classification performance for the minority class [38]. Below are some significant limitations associated with imbalanced datasets:

- Biased model performance: Models perform poorly on the minority class while frequently predicting the majority class.
- Poor recall and precision: The minority class often suffers from low recall and precision.
- Overfitting on the majority class: The model may overfit the majority class, further degrading overall performance.
- Difficulty distinguishing noise from minority examples: Classifiers may struggle to differentiate between noise and valid minority class instances, often ignoring the minority class entirely [39].

In the case of credit card fraud detection, datasets are typically imbalanced because fraudulent transactions occur rarely compared to a large number of legitimate transactions. To address this issue, various oversampling techniques can be employed, such as Random Oversampling, SMOTE, Borderline SMOTE, ADASYN, SMOTE-ENN, and SMOTE-NC. In this study, we apply the SMOTE (Synthetic Minority Over-sampling Technique) approach when the ratio between genuine and fraudulent transactions exceeds 85% to 30%. For the highly imbalanced dataset (ECC-2013), where we apply both SMOTE and the advanced oversampling technique ADASYN, SMOTE-ENN.

The primary concept of SMOTE is to generate synthetic instances for the minority class by interpolating between multiple adjacent minority class examples. This helps mitigate the issue of overfitting and allows the decision boundaries for the minority class to extend further into the majority class domain [39]. SMOTE achieves this by oversampling the minority class, creating synthetic examples along the line segments that connect each minority class sample with its *k*-nearest neighbors (KNN) within the same class. Neighbors are randomly selected based on the required level of oversampling. The synthetic samples are generated as follows: (i) Calculate the difference between the feature vector of the current sample and its nearest neighbor. (ii) Multiply this difference by a random number in the range of 0 to 1. (iii) Add the result to the original feature vector. This process generates a new data point at a random location along the line segment connecting the two feature vectors. By doing so, SMOTE effectively expands the decision region for the minority class, reducing bias and improving classification performance [39].

$$x_{\text{new}}=x_{i}+(x_{i}^{\prime }-x_{i})\cdot \alpha ,$$ (1)

where $x_{i}^{\prime }$ is one of the K-nearest neighbors of *x*_*i*_, and α∈[0,1] is a real random number.

SMOTE-ENN is a hybrid resampling technique that performs both oversampling and undersampling of the data. Here, the first SMOTE technique is to oversample the minority class and then undersample the edited nearest neighbor (ENN) to remove the overlapping instances to obtain a balanced dataset [40]. Building upon SMOTE, ADASYN enhances synthetic sample generation for the minority class by adaptively focusing on regions where the classifier struggles due to class imbalance. By generating more synthetic samples in areas with higher classification complexity, ADASYN dynamically adjusts the data distribution, improving the overall learning process [41].

#### 4.1.2 Encoding.

Machine learning algorithms require numerical inputs; thus, it is essential to convert categorical variables into numerical values using encoding techniques. Various encoding methods are available to perform this conversion, including one-hot encoding, ordinal encoding, sum coding, Helmert coding, polynomial coding, backward difference coding, and binary coding [42]. In this paper, we employed the one-hot encoding technique to encode categorical variables. One-hot encoding is one of the most widely used encoding schemes. It compares the levels of a categorical variable to a predefined reference level. This method transforms a single variable with *n* observations and *d* unique values into *d* binary variables, each containing *n* observations. Each binary variable indicates the presence (1) or absence (0) of a specific category [42].

#### 4.1.3 Normalization.

Normalization involves transforming data values to a specific range, such as 0 to 1 or −1 to 1. This technique is particularly beneficial for mining tasks like classification, artificial neural networks (ANN), and clustering algorithms. It is especially useful in backpropagation neural networks, where scaling the data properties can significantly accelerate the learning process. Common normalization methods include min-max normalization, z-score normalization, and decimal scaling [36]. In this study, we applied min-max normalization to both training and testing samples, which is applied for effective feature scaling [43]. This method converts numerical feature values into a new range (0 to 1) based on their minimum and maximum values [44]. Min-max normalization rescales the values of a feature using Equation (1):

$$X_{\text{scaled}}=\frac{X-X_{\text{MIN}}}{X_{\text{MAX}}-X_{\text{MIN}}},$$ (2)

where *X* is the original feature value, $X_{\text{MIN}}$ and $X_{\text{MAX}}$ are the minimum and maximum values, respectively, in the dataset, and $X_{\text{scaled}}$ is the normalized value of the feature.

### 4.2 Feature selection

Feature selection (FS) is an essential phase in the implementation of machine learning techniques, as it enables the extraction of the most relevant attributes for accurate classification [45]. This is mainly due to the dataset employed during the training and testing phases potentially possessing a vast feature space, which may negatively affect the models’ overall performance [7]. The following papers use different FS techniques to develop their ML models. Kasongo [46], Ileberi *et al*. [7], and Hassanat *et al*. [47] employ genetic algorithm-based FS techniques to enhance the performance of machine learning models. Mienye *et al*. [48] implemented a particle swarm optimization (PSO) technique for their heart disease prediction. Will Koehrsen developed a feature selector tool [49], which Varmedja *et al*. [50] used to detect credit card fraud. Bhowmik *et al*. [51] implemented a hybrid feature selection technique for phishing website prediction. In the paper [52], the authors propose a GA-KNN feature selection method that identifies optimal feature combinations and enhances overall model performance. For imbalanced datasets, this paper [53] proposes a two-stage approach integrating enhanced Sparse Autoencoder (SAE) for feature learning and Softmax regression for classification, aiming to improve minority class prediction performance. In this paper [54], the authors proposed an unsupervised feature learning method using a stacked Sparse Autoencoder (SSAE). The SSAE learn robust feature representations that were employed to train classifiers and enhance performance. The authors of this paper [40], proposed a hybrid resampling technique that combines both undersampling using Edited Nearest Neighbor (ENN) and oversampling using the SMOTE technique (SMOTE-ENN) to balance the dataset. In this paper [55], the authors proposed a feature selection technique that combines Information Gain with a cost-sensitive Adaptive Boosting (AdaBoost) classifier for chronic kidney disease prediction.

In this paper, we implement a hybrid feature selection technique. This approach integrates two distinct methodologies: the filter and the embedding methods. The initial phase of this hybrid strategy involves the filter method, which incorporates two techniques utilized in this paper: Pearson correlation and Information Gain. We first employ Pearson correlation on the balanced dataset to determine highly correlated features, as the SMOTE technique generates synthetic instances that enhance the correlations among features. Synthetic instances are likely to reside within the same region of the feature space when generated along the line segment connecting a minority class instance to its nearest neighbor. As a result, the characteristics of these synthetic cases may exhibit strong correlations with each other. The Pearson Correlation Coefficient has a range of values from -1 to 1. A value of -1 indicates a negative correlation between the data, a value of 1 indicates a positive correlation and a value of 0 indicates no correlation exists between the variables [69]. This paper employs a Pearson correlation threshold of ± 0.95 to identify highly correlated features. Features exceeding this threshold are removed, leaving only the uncorrelated features (F1).

Secondly, we apply two filtering methods, Information Gain (IG) and the embedded Random Forest Importance (RFI) technique, on the features (F1). Each method calculates an importance score for every feature and has its own threshold value. In this paper, we manually selected different optimized threshold values for each dataset because different datasets have varying characteristics, such as data imbalance ratios, feature distributions and relationships, feature dimensionality, and model complexity. Features from both methods that exceed their respective threshold values are stored in separate sets: F2 for IG-selected features and F3 for RFI-selected features. Finally, the union of F2 and F3 (F) contains all features selected by either method.

$$F=F_{2}\cup F_{3}.$$ (3)

This ensures that features selected by at least one of the methods are included in the final feature set (F).

**Algorithm 2. Hybrid feature selection.**

**Input:** Balanced Dataset ($Tx_{\text{SMOTE}}$, $Ty_{\text{SMOTE}}$)

**Output:** Final Selected Features (F)

**Step 1: Pearson Correlation**

1: Compute Pearson correlation for all feature pairs in $Tx_{\text{SMOTE}}$

2: $F_{1}\leftarrow \{f_{i}\in Tx_{\text{SMOTE}}||\text{PearsonCorrelation}(f_{i},f_{j})|<0.95\;\forall j\neq i\}$    ⊳ Remove highly correlated features based on threshold ±0.95

**Step 2: Information Gain (IG)**

3: Compute Information Gain scores for all selected features in *F*_1_

4: Select threshold $TH_{\text{IG}}$

5: $F_{2}\leftarrow \{f_{i}\in F_{1}|\text{IG}(f_{i})\geq TH_{\text{IG}}\}$    ⊳ Select features exceeding the threshold

**Step 3: Random Forest Importance (RFI)**

6: Compute Random Forest Importance scores for all features in *F*_1_

7: Select threshold $TH_{\text{RFI}}$

8: $F_{3}\leftarrow \{f_{i}\in F_{1}|\text{RFI}(f_{i})\geq TH_{\text{RFI}}\}$ ⊳ Select features exceeding the threshold

**Step 4: Combine Selected Features**

9: $F\leftarrow F_{2}\cup F_{3}$ ⊳ Union of features selected by IG and RFI

10: **Return** *F*

After using Pearson Correlation, RFI, and IG to choose features, Tables 4, 5, 6, 7 and 8 show how many features are in each dataset: European Cardholders 2013, European Cardholders 2023, the Australian Dataset, the Abstract Dataset, and the German Dataset respectively. After applying the SMOTE technique, European Cardholders 2013 and the Abstract Dataset remove correlated features illustrated in Table 4 and Table 7, respectively. Table 4 shows that we chose V16 for the Pearson correlation method and took it out of the dataset because it had a strong correlation with V17 and was higher than the chosen threshold value of 0.95. Table 7, using the same feature selection technique, reveals a high correlation between the *6_month_avg_chbk_amt* and *Daily_chargeback_avg_amt* features. In this case, remove only Daily_chargeback_avg_amt features from the dataset. Table 5 shows that no features were removed from the European Cardholders 2023 dataset using the Pearson correlation method, as none of the feature pairs exceeded the correlation threshold. Similarly, Table 6 indicates that the Australian Dataset retained all features during Pearson correlation analysis. Finally, Table 8 confirms that the German Dataset also did not require the removal of any features using this method, as all correlations remained below the threshold.

**Table 4:** Feature selection from the European Cardholders 2013.

Resampling Techniques	Feature Selection Method	Threshold level	No. of Features	Selected Features
SMOTE	PC (F1)	0.95	1	‘V16’
	RFI (F2)	0.0105	14	‘V14’, ‘V10’, ‘V17’, ‘V12’, ‘V4’, ‘V11’, ‘V3’, ‘V7’, ‘V9’, ‘V2’, ‘V18’, ‘V1’, ‘V21’, ‘V6’
	IG (F3)	0.183	17	‘V14’, ‘V12’, ‘V10’, ‘V4’, ‘V17’, ‘V11’, ‘Amount’, ‘V3’, ‘V7’, ‘V2’, ‘V9’, ‘Time’, ‘V27’, ‘V21’, ‘V1’, ‘V18’, ‘V6’
	F=F2∪F3	-	17	$\overset{`}{\text{V}}$14’, ‘V12’, ‘V10’, ‘V4’, ‘V17’, ‘V11’, ‘Amount’, ‘V3’, ‘V7’, ‘V2’, ‘V9’, ‘Time’, ‘V27’, ‘V21’, ‘V1’, ‘V18’, ‘V6’
SMOTE-ENN	PC (F1)	0.95	1	‘V16’
	RFI (F2)	0.0105	15	‘V14’, ‘V10’, ‘V12’, ‘V17’, ‘V4’, ‘V11’, ‘V3’, ‘V7’, ‘V2’, ‘V9’,‘V18’, ‘V21’, ‘V1’, ‘V27’, ‘V6’
	IG (F3)	0.183	17	‘V14’, ‘V12’, ‘V10’, ‘V4’, ‘V17’, ‘V11’, ‘V3’, ‘Amount’, ‘V7’,‘V2’, ‘Time’, ‘V9’, ‘V27’, ‘V21’, ‘V1’, ‘V18’, ‘V6’
	F=F2∪F3	-	17	‘V11’, ‘V6’, ‘V21’, ‘V1’, ‘V14’, ‘V7’, ‘V10’, ‘V18’, ‘V27’, ‘V4’, ‘V12’, ‘V17’, ‘V9’, ‘V3’, ‘Amount’, ‘V2’, ‘Time’
ADASYN	PC (F1)	0.95	0	-
	RFI (F2)	0.0105	13	‘V14’, ‘V12’, ‘V4’, ‘V10’, ‘V17’, ‘V11’, ‘V16’, ‘V3’, ‘V18’, ‘V9’,‘V6’, ‘V2’, ‘V7’
	IG (F3)	0.183	17	‘V14’, ‘V12’, ‘V10’, ‘V4’, ‘V17’, ‘Amount’, ‘V11’, ‘V3’, ‘V16’,‘V7’, ‘V2’, ‘V9’, ‘Time’, ‘V27’, ‘V21’, ‘V1’, ‘V18’
	F=F2∪F3	-	18	$\overset{`}{\text{V}}$10’, ‘V12’, ‘V18’, ‘V2’, ‘V16’, ‘V1’, ‘V11’, ‘V6’, ‘V7’, ‘Time’, ‘V17’, ‘V21’, ‘V14’, ‘V3’, ‘V9’, ‘V4’, ‘V27’, ‘Amount’

**Table 5:** Feature selection from the European Cardholders 2023.

Feature Selection Method	Threshold level	No. of Features	Selected Features
PC (F1)	0.95	0	-
RFI (F2)	0.0105	14	$\overset{`}{\text{V}}$14’, ‘V12’, ‘V10’, ‘V17’, ‘V4’, ‘V11’, ‘V16’, ‘V7’, ‘V2’, ‘V3’,‘V18’, ‘V9’, ‘V1’, ‘V21’
IG (F3)	0.183	16	$\overset{`}{\text{V}}$14’, ‘V17’, ‘V10’, ‘V12’, ‘V4’, ‘V11’, ‘V3’, ‘V16’, ‘V7’, ‘V2’,‘V9’, ‘V21’, ‘V27’, ‘V18’, ‘V1’, ‘V6’
F=F2∪F3	-	16	$\overset{`}{\text{V}}$2’, ‘V4’, ‘V9’, ‘V1’, ‘V11’, ‘V18’, ‘V16’, ‘V12’, ‘V10’, ‘V14’, ‘V21’, ‘V7’, ‘V27’, ‘V17’, ‘V3’, ‘V6’

**Table 6:** Feature selection from the Australian dataset.

Feature Selection Method	Threshold level	No. of Features	Selected Features
PC (F1)	0.95	0	-
RFI (F2)	0.0065	14	À8’, ‘A10’, ‘A7’, ‘A14’, ‘A3’, ‘A5’, ‘A2’, ‘A13’, ‘A9’, ‘A6’, ‘A4’, ‘A1’, ‘A11’, ‘A12’
IG (F3)	0.02	8	À8’, ‘A10’, ‘A9’, ‘A14’, ‘A7’, ‘A5’, ‘A3’, ‘A12’
F=F2∪F3	-	14	À14’, ‘A8’, ‘A5’, ‘A1’, ‘A13’, ‘A9’, ‘A11’, ‘A7’, ‘A4’, ‘A12’, ‘A2’, ‘A10’, ‘A6’, ‘A3’

**Table 7:** Feature selection from the Abstract dataset.

Feature Selection Method	Threshold level	No. of Features	Selected Features
PC (F1)	0.95	1	‘Daily_chargeback_avg_amt’
RFI (F2)	0.0105	7	‘Total Number of declines/day’, ‘Transaction_amount’, ‘6_month_avg_chbk_amt’, ‘isForeignTransaction’, ‘6-month_chbk_freq’, ‘isHighRiskCountry’, ‘Average Amount/transaction/day’
IG (F3)	0.0183	8	‘Total Number of declines/day’, ‘Transaction_amount’, ‘Is declined’, ‘6_month_avg_chbk_amt’, ‘isForeignTransaction’, ‘6-month_chbk_freq’, ‘isHighRiskCountry’, ‘Average Amount/transaction/day’
F=F2∪F3	-	8	‘Total Number of declines/day’, ‘Transaction_amount’, ‘Is declined’, ‘6_month_avg_chbk_amt’, ‘isForeignTransaction’, +6-month_chbk_freq’, ‘isHighRiskCountry’, ‘Average Amount/transaction/day’

**Table 8:** Feature selection from the German dataset.

Feature Selection Method	Threshold level	No. of Features	Selected Features
PC (F1)	0.95	0	-
RFI (F2)	0.0065	47	‘age’, ‘credithistory_A32’, ..., ‘credithistory_A34’, ‘creditamount’, ’duration’, ‘employmentsince_A71’, ..., ‘employmentsince_A75’, ‘existingchecking_A11’, ..., ‘existingchecking_A14’, ‘existingcredits’, ‘housing_A151’, ‘housing_A152’, ‘installmentrate’, ’job_A172’, ..., ’job_A174’, ‘otherdebtors_A101’, ‘otherdebtors_A103’, ‘otherinstallmentplans_A141’, ‘otherinstallmentplans_A143’, ‘peopleliable’, ‘property_A121’, ..., ‘property_A124’, ‘purpose_A40’, ..., ‘purpose_A43’, ‘purpose_A49’, ‘residencesince’, ‘savings_A61’, ..., ‘savings_A65’, ‘statussex_A92’, ..., ‘statussex_A94’, ‘telephone_A191’, ‘telephone_A192’
IG (F3)	0.018	20	‘age’, ‘creditamount’, ‘credithistory_A30’, ‘credithistory_A34’, ’duration’, ‘employmentsince_A75’, ‘existingchecking_A12’, ‘existingchecking_A14’, ‘housing_A152’, ’job_A172’, ‘otherdebtors_A102’, ‘otherinstallmentplans_A143’, ‘property_A121’, ‘purpose_A40’, ‘purpose_A43’, ‘purpose_A49’, ‘residencesince’, ‘savings_A63’, ‘savings_A64’, ‘statussex_A93’
F=F2∪F3	-	49	‘age’, ‘credithistory_A32’, ..., ‘credithistory_A34’, ‘creditamount’, ’duration’, ‘employmentsince_A71’, ..., ‘employmentsince_A75’, ‘existingchecking_A11’, ..., ‘existingchecking_A14’, ‘existingcredits’, ‘housing_A151’, ‘housing_A152’, ‘installmentrate’, ’job_A172’, ..., ’job_A174’, ‘otherdebtors_A101’, ‘otherdebtors_A103’, ‘otherinstallmentplans_A141’, ‘otherinstallmentplans_A143’, ‘peopleliable’, ‘property_A121’, ..., ‘property_A124’, ‘purpose_A40’, ..., ‘purpose_A43’, ‘purpose_A49’, ‘residencesince’, ‘savings_A61’, ..., ‘savings_A65’, ‘statussex_A92’, ..., ‘statussex_A94’, ‘telephone_A191’, ‘telephone_A192’

For the IG feature selection technique, the total number of features across the five datasets is 17, 16, 8, 8, and 20, respectively. Similarly, for the RFI feature selection, the total number of features is 14, 14, 14, 7, and 47, respectively. Finally, use the set union operation on the features to combine all the unique features from both methods. This will give the total number of features for these five datasets, which are 17, 16, 14, 8, and 49, respectively.

### 4.3 Ensemble learning

**Fig 4:**
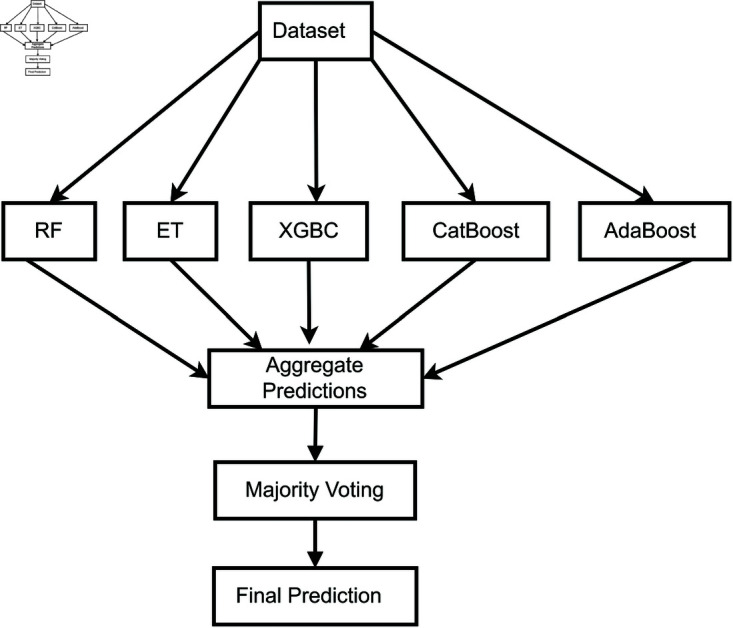
Ensemble majority voting.

Ensemble learning is a method that combines multiple ML classifier models to achieve improved performance compared to the individual classifier model. The predictions from individual models are aggregated through a combination rule to produce a more accurate single prediction, rather than depending on a single model [56]. Ensemble models can outperform individual base learners even if some base learners are weak. The performance of the ensemble learning depends mainly on the accuracy and diversity of the base learners [57]. A crucial step in constructing ensemble classifiers is to combine the individual base learners. The ensemble learning method typically determines the combination mechanism employed. The most popular mechanism is the majority vote to combine ensemble base models, as shown in Fig 4.

**Algorithm 3. Ensemble classification with majority voting (hard voting).**

**Input:** Training Data $\left(X_{\text{train}},Y_{\text{train}}\right)$, Test Data $X_{\text{test}}$

**Output:** Predicted Class Labels ${\hat{Y}}_{\text{ensemble}}$

1: Models←[RandomForestClassifier(),ExtraTreesClassifier(),XGBClassifier(), AdaBoostClassifier(),CatBoostClassifier()]

2: **for each** model *M* in Models **do**    ⊳ Train each model on the training data

3:   $M.\text{fit}(X_{\text{train}},Y_{\text{train}})$

4: **end for**

5: $\hat{Y}\leftarrow \text{empty list}$    ⊳ Initialize list to store predictions

6: **for each** model *M* in Models **do**

7:   $\hat{Y}\leftarrow M.\text{predict}(X_{\text{test}})$    ⊳ Generate predictions on the test data

8: **end for**

9: **for each** instance *i* in $X_{\text{test}}$ **do**

10:   $V_{i}\leftarrow [{\hat{Y}}_{j}[i]\text{ for each }{\hat{Y}}_{j}\text{ in }\hat{Y}]$

11:   ${\hat{Y}}_{\text{ensemble}}[i]\leftarrow Mode(V_{i})$    ⊳ Most Common Class

12: **end for**

13: **Return** ${\hat{Y}}_{\text{ensemble}}$

In a classification problem, the predictions for each class are aggregated, and the class with the majority vote is identified as the ensemble prediction. In regression tasks, the majority vote is obtained by calculating the average predictions from multiple base learners [58]. Let the decision of the *t*-th classifier be $d_{t,c}\in \{0,1\}$, where, t=1,…,T and c=1,…,C. Then, using majority voting, the class $\omega_{c^{*}}$ is selected as the ensemble prediction such that


$$c^{*}=\arg\max_{c}\sum_{t=1}^{T}d_{t,c},$$

where *T* is the number of classifier models and *C* represents the number of classes.

Diverse classifiers tend to make uncorrelated errors, and by aggregating their predictions, the ensemble can mitigate individual weaknesses and reduce variance. In our case, combining tree-based models such as RF, ET, XGBC, AdaBoost, and CatBoost—each with different internal learning mechanisms—introduced sufficient diversity to enhance generalization. This aligns with ensemble theory, which states that a set of accurate and diverse models will yield a more robust and stable prediction than any single model alone. Therefore, ensemble voting not only combines predictions but also leverages the complementary strengths of heterogeneous classifiers.

## 5 Evaluation metrics

Model evaluation holds significant importance in predictive modeling tasks. In ensemble predictive modeling, the evaluation of relative performance and model diversity is essential. Evaluation metrics are derived from four classifications: true positives (TP), true negatives (TN), false positives (FP), and false negatives (FN) [59].

This paper presents research that is structured as a binary classification task in machine learning. As a result, the accuracy (AC) derived from the test data serves as the primary performance metric. Furthermore, for each model, the recall (RC), precision (PR), and F1-Score (F-Measure) are calculated [60]. To evaluate the classification quality of each model, we additionally plot the Area Under the Curve (AUC) and Precision Recall Curve (PR).

### 5.1 Accuracy

Accuracy is commonly employed to evaluate the performance of a model utilizing the confusion matrix [59].

$$\text{Accuracy}=\frac{TP+TN}{TP+TN+FN+FP}.$$ (4)

### 5.2 Precision

Precision is defined as the ratio of true positives to the sum of true positives and false positives. In this context, precision quantifies the proportion of instances identified as positive by the classifier that are truly positive [59].

$$\text{Precision}=\frac{TP}{TP+FP}.$$ (5)

### 5.3 Recall

Recall is defined as the ratio of true positives to the sum of true positives and false negatives [59].

$$\text{Recall}=\frac{TP}{TP+FN}.$$ (6)

### 5.4 F1 Score

The F1-Score is a metric that incorporates both Precision and Recall to validate accuracy. It is the harmonic mean of Precision and Recall [60]. The relationship between precision and recall involves a trade-off: higher precision is typically associated with lower recall [59]. The harmonic mean of Recall and Precision is defined as follows:

$$\text{F1 Score}=\frac{2\cdot \text{Precision}\cdot \text{Recall}}{\text{Precision}+\text{Recall}}.$$ (7)

### 5.5 Areas under the receiver operating characteristic (ROC) curve

ROC curve plots the False Positive Rate (FPR) on the X-axis and the True Positive Rate (TPR) on the Y-axis. FPR measures the fraction of negative examples that are misclassified as positive, while TPR measures the fraction of positive examples that are correctly labeled [61]. The Area Under the Curve (AUC) provides a summary of the ROC curve, with values that range from 0 to 1. An AUC of 0 indicates that the model’s predictions are entirely incorrect, whereas an AUC of 1 signifies that all predictions made by the model are accurate [62]. AUC measures the overall performance of a test; the better the AUC score, the better the overall performance [63].

### 5.6 Precision recall curve

The Precision-Recall (PR) curve plots Recall on the X-axis and Precision on the Y-axis. Recall is equivalent to the True Positive Rate (TPR), while Precision quantifies the proportion of instances identified as positive that are truly positive [61].

### 5.7 Matthews Correlation Coefficient (MCC)

Using a single indicator MCC provides the greatest description of true and false positives and negatives. It evaluates a two-class problem’s quality by considering both true and false positives as well as negatives. There is a balanced measurement when the class sizes are varied [64]. The equation of MCC is as follows

$$\text{MCC}=\frac{TP\times TN-FP\times FN}{\sqrt{\left(TP+FP)(TP+FN)(TN+FP)(TN+FN\right)}}$$ (8)

## 6 Result and discussion

**Fig 5:**
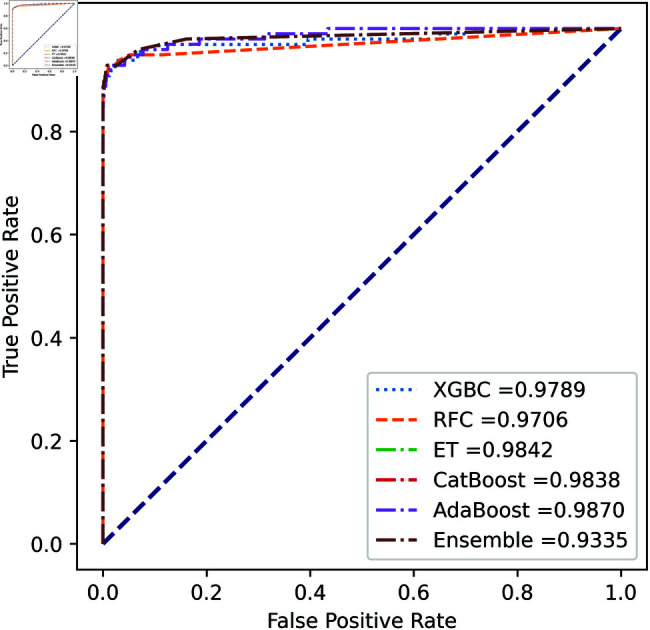
ROC Curve for European cardholder 2013 (SMOTE).

**Fig 6:**
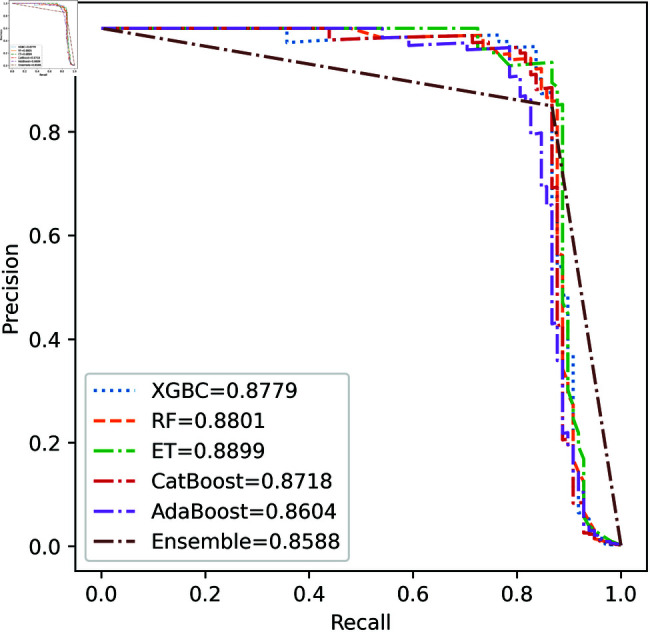
PR Curve for European cardholder 2013 (SMOTE).

**Fig 7:**
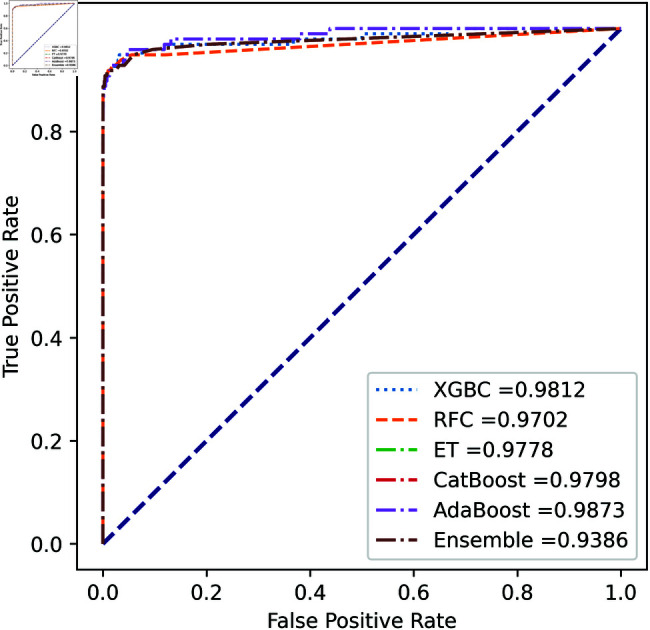
ROC Curve for European cardholder 2013 (SMOTE-ENN).

**Fig 8:**
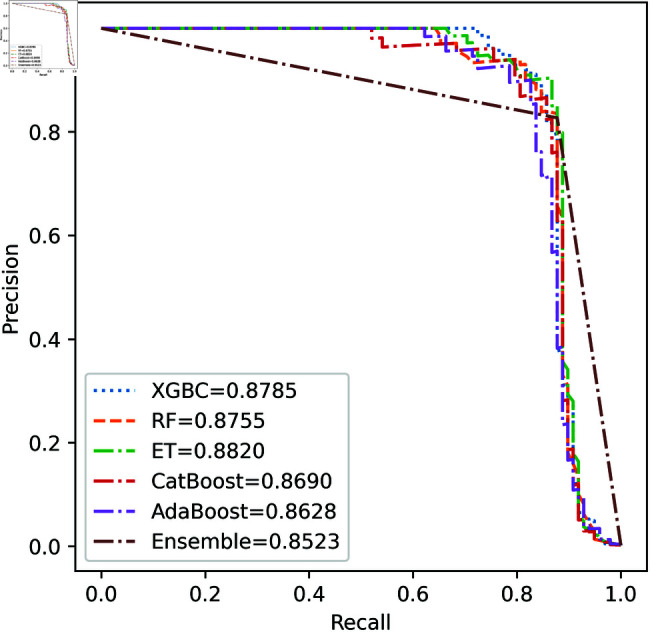
PR Curve for European cardholder 2013 (SMOTE-ENN).

**Fig 9:**
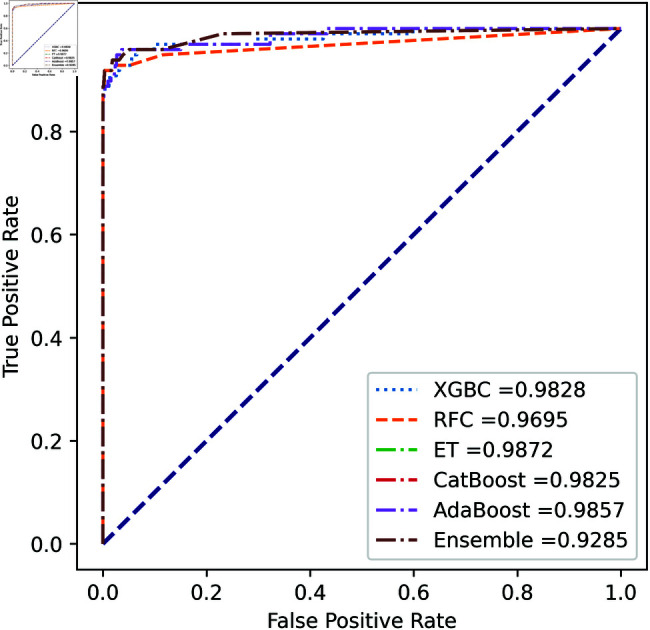
ROC Curve for European cardholder 2013 (ADASYN).

**Fig 10:**
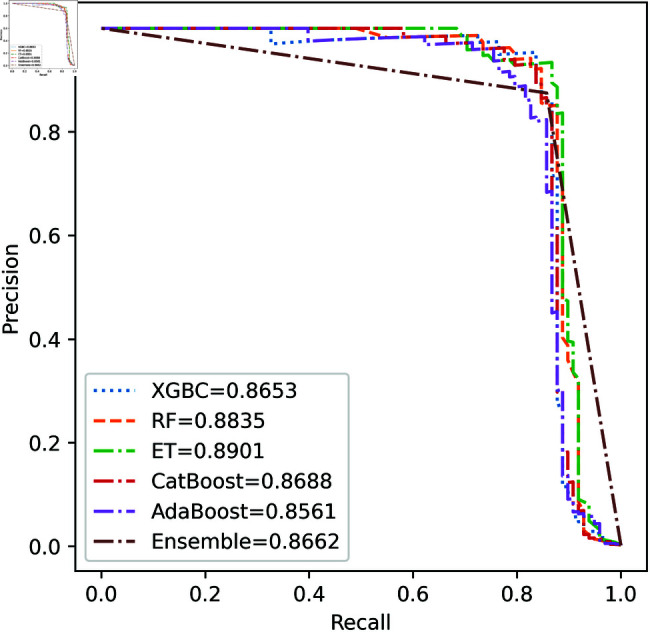
PR Curve for European cardholder 2013 (ADASYN).

**Fig 11:**
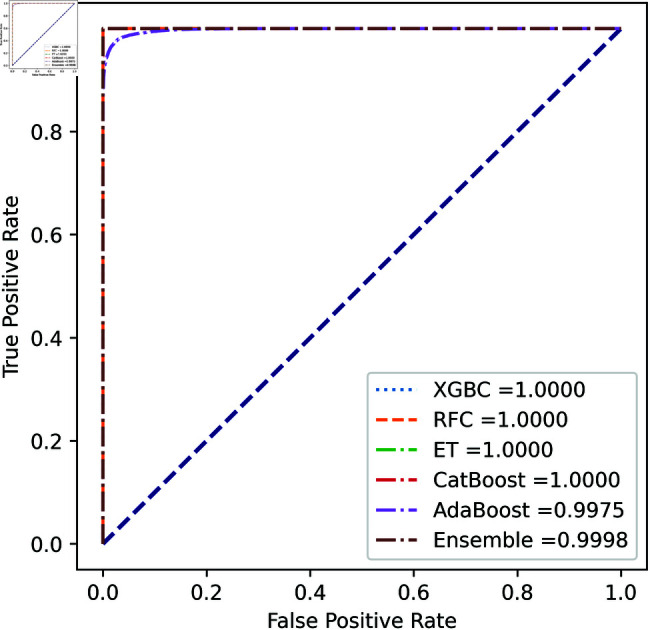
ROC Curve for European cardholder 2023.

**Fig 12:**
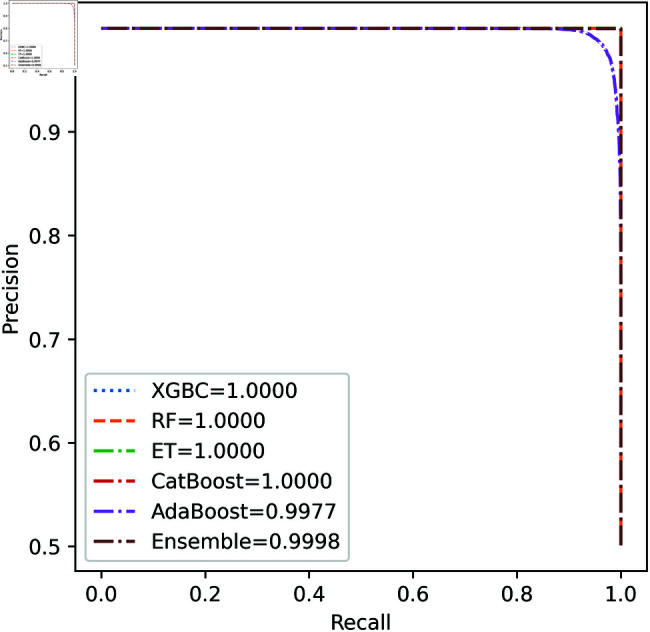
PR Curve for European cardholder 2023.

**Fig 13:**
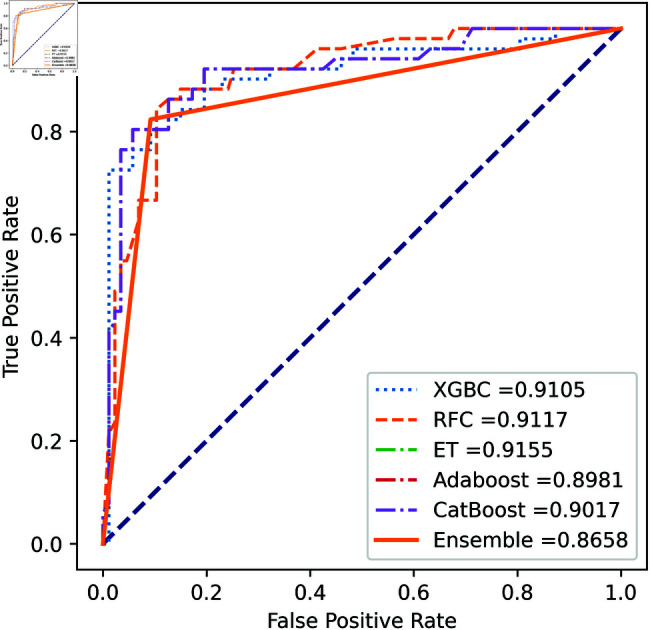
ROC Curve for Australian dataset.

**Fig 14:**
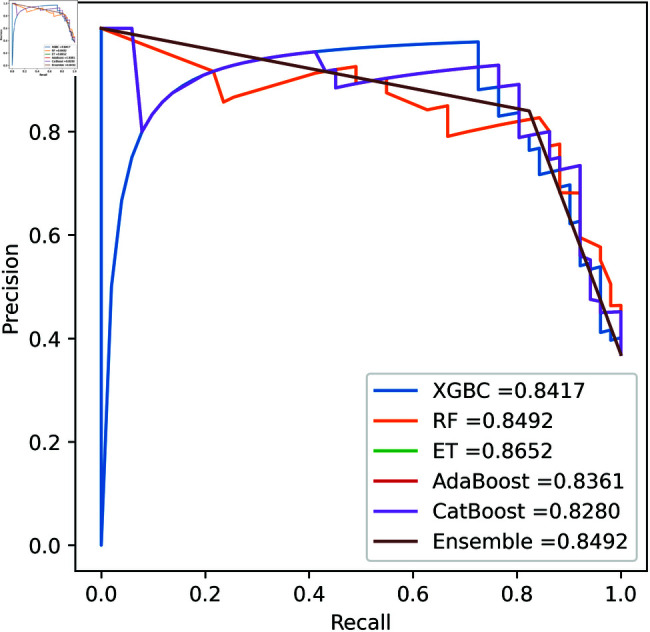
PR Curve for Australian dataset.

**Fig 15:**
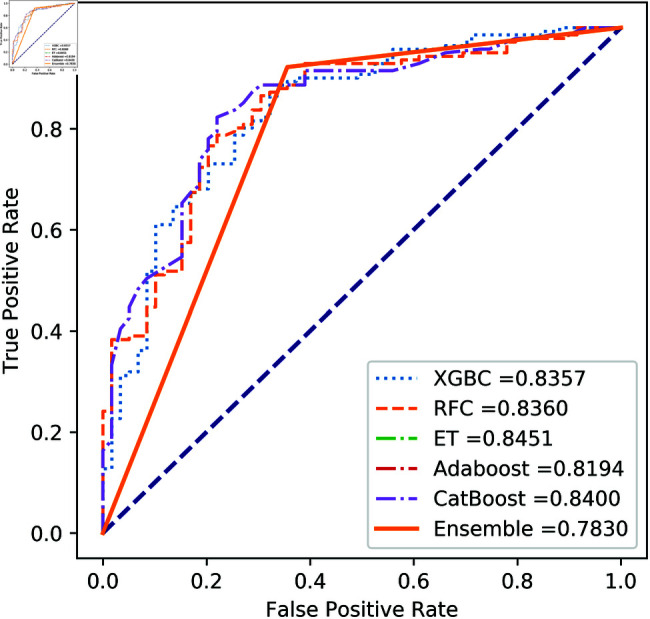
ROC Curve for German dataset.

**Fig 16:**
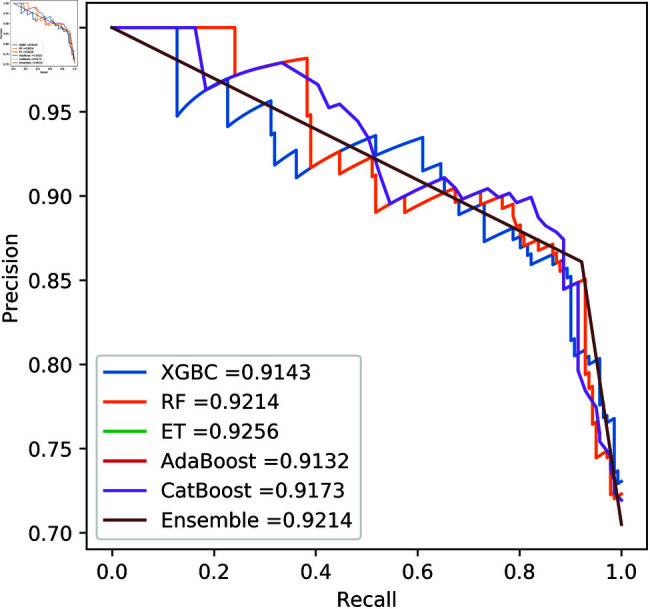
PR Curve for German dataset.

**Fig 17:**
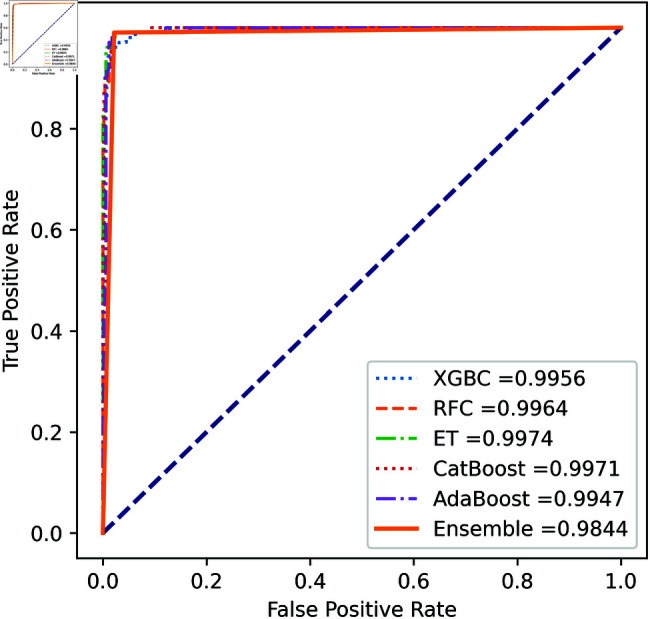
ROC Curve for Abstract dataset.

**Fig 18:**
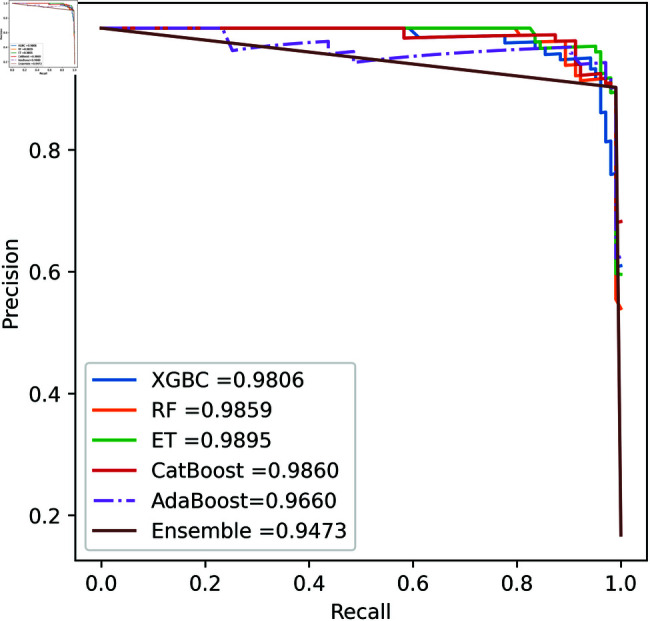
PR Curve for Abstract dataset.

We apply five ML model classifiers to five different datasets to validate our proposed methodology, including RF, XGBC, ET, AdaBoost, CatBoost, and the ensemble learning method described in Sect 4.2. The results are summarized in Tables 9, 10, 11, 12 and 13 and Figs 5–18. The evaluation of a model’s performance is based on its accuracy, precision, recall, f1 score, AUROC, PR score, and MCC. The best score for each model is in bold. As described in Sect 4.1.1, we applied SMOTE, SMOTE-ENN, and ADASYN techniques to address class imbalance in the dataset. Table 9 and Figs 5-10 show the performance of various models using these oversampling techniques. Among the five classifier models tested across these techniques, the Extra Trees (ET) classifier consistently outperformed the others. It achieved an accuracy of 99.97% with SMOTE and ADASYN, and 99.96% with SMOTE-ENN. The F1 scores for SMOTE and ADASYN with the ET model were also identical. Notably, the ADASYN-ET combination achieved the highest AUC (98.72%) and MCC (89.99%) scores. Therefore, for this dataset, our proposed model is ADASYN-ET.

**Table 9:** Classification performance measures for the European Cardholders 2013.

Resampling Technique	Models	Accuracy (%)	Precision (%)	Recall (%)	F1 Score (%)	AUC (%)	PR (%)	MCC (%)
SMOTE	RF	99.95	85.85	86.73	86.29	97.06	88.01	85.99
	XGBC	99.95	85.86	86.73	86.29	97.89	87.79	85.69
	ET	**99.97**	**93.41**	86.73	**89.95**	98.42	**88.99**	**88.85**
	CatBoost	99.92	75.89	86.73	80.95	98.38	87.18	81.09
	AdaBoost	99.35	19.77	**89.79**	32.41	**98.70**	86.04	47.75
	Ensemble Vote	99.95	84.16	86.73	85.43	93.35	85.88	86.58
SMOTE-ENN	RF	99.95	84.0	85.71	84.84	97.02	87.55	84.82
	XGBC	99.94	79.62	87.75	83.50	98.12	87.85	83.56
	ET	**99.96**	**89.47**	86.73	**88.08**	97.78	**88.20**	**88.07**
	CatBoost	99.93	77.27	86.73	81.73	97.98	86.90	81.83
	AdaBoost	99.54	25.82	87.75	39.90	**98.73**	86.28	47.47
	Ensemble Vote	99.95	82.69	**87.76**	85.14	93.86	85.23	85.16
ADASYN	RF	99.95	87.36	84.69	86.01	96.95	88.35	85.99
	XGBC	99.95	85.71	85.71	85.71	98.28	86.53	86.69
	ET	**99.97**	**93.41**	86.73	**89.95**	**98.72**	**89.01**	**89.99**
	CatBoost	99.92	75.89	86.73	80.95	98.25	86.88	81.09
	AdaBoost	99.55	26.13	**87.75**	40.28	98.57	85.61	47.75
	Ensemble Vote	99.95	87.5	85.71	86.59	92.85	86.62	86.58

**Table 10:** Classification performance measures for the European Cardholders 2023.

Models	Accuracy (%)	Precision (%)	Recall (%)	F1 Score (%)	AUC (%)	PR (%)	MCC (%)
RF	99.98	99.97	99.99	99.98	**100**	**100**	99.97
XGBC	99.97	99.94	**100**	99.97	**100**	**100**	99.95
ET	**99.99**	**99.98**	**100**	**99.99**	**100**	**100**	**99.98**
CatBoost	99.94	99.89	**100**	99.94	**100**	**100**	99.87
AdaBoost	97.43	98.33	96.52	97.41	99.75	99.77	94.89
Ensemble Vote	99.98	99.97	**100**	99.98	99.98	99.98	99.97

**Table 11:** Classification performance measures for the Australian dataset.

Models	Accuracy (%)	Precision (%)	Recall (%)	F1 Score (%)	AUC (%)	PR (%)	MCC (%)
RF	85.51	81.63	78.43	80.00	91.17	84.92	71.69
XGBC	86.23	82.00	80.39	81.19	91.05	84.17	68.89
ET	89.13	**89.13**	80.39	84.53	**91.55**	**86.52**	71.63
CatBoost	85.50	81.63	78.43	80.00	89.81	83.61	67.01
AdaBoost	**89.86**	**86.27**	**86.27**	**86.27**	90.17	82.80	**78.22**
Ensemble Vote	87.68	84.00	82.35	83.16	86.58	84.92	70.34

**Table 12:** Classification performance measures for the German dataset.

Models	Accuracy (%)	Precision (%)	Recall (%)	F1 Score (%)	AUC (%)	PR (%)	MCC (%)
RF	83.5	85.06	**92.91**	88.81	83.60	92.14	46.53
XGBC	82.0	85.23	90.70	87.58	83.57	91.43	49.56
ET	83.0	**87.41**	88.65	88.02	**84.51**	**92.56**	47.68
CatBoost	80.5	84.93	87.94	86.41	81.94	91.73	47.68
AdaBoost	80.5	85.42	87.23	86.31	84.00	91.32	47.64
Ensemble Vote	**84.0**	86.09	92.20	**89.04**	78.30	92.14	**50.59**

**Table 13:** Classification performance measures for the Abstract dataset.

Models	Accuracy (%)	Precision (%)	Recall (%)	F1 Score (%)	AUC (%)	PR (%)	MCC (%)
RF	96.59	83.61	**99.03**	90.67	99.64	98.59	89.99
XGBC	97.72	**91.59**	95.15	93.33	99.56	98.06	91.16
ET	96.10	81.60	**99.03**	89.47	99.74	**98.95**	88.16
CatBoost	97.72	90.83	96.11	93.40	**99.71**	98.60	92.69
AdaBoost	**98.05**	90.99	98.06	94.39	99.47	96.60	**93.30**
Ensemble Vote	**98.05**	90.26	99.02	**94.44**	98.44	94.73	92.28

For the European Cardholders 2023 dataset, ET also provides superior performance with accuracy; the f1 score, AUC, PR score, and MCC are 99.99%, 99.99%, 100%, 100%, and 99.98% respectively, as shown in Table 10 and Figs 11 and 12. In this dataset, all models perform well, with the exception of AdaBoost, which has a lower accuracy score of 97.43% compared to the other models. For the Australian dataset, AdaBoost provides superior performance with an accuracy score of 89.86%, 86.27% of the f1 score, and 78.22% MCC score. In this dataset, ET provides the highest AUC and PR scores of 91.55% and 86.52%, respectively. For the German dataset, the performance scores presented in Table 12 show that ensemble majority voting achieved the highest accuracy, MCC, and F1 score, reaching 84%, 50.59%, and 89.04%, respectively. For the Abstract dataset, the performance scores are presented in Table 13 and Figs 17 and 18. These results indicate that both AdaBoost and ensemble majority voting achieved the highest accuracy score of 98.05%, while the F1 score was 0.05% higher for ensemble learning. Figs 17 and 18 also show that the Extra Trees (ET) model achieved the highest AUC and PR scores, at 99.74% and 98.95%, respectively. The above discussion shows that our proposed hybrid feature selection technique performs effectively across all classifier models.

### 6.1 Statistical analysis

To assess the statistical significance of the performance metrics, we applied a one-way Analysis of Variance (ANOVA). This test determines whether there are statistically significant differences between the means of multiple groups. Null hypothesis significance testing was used to support the interpretation of results and to ensure that claims of improved performance are supported by statistical evidence. A p-value less than the predefined significance level (0.05) indicates a statistically significant result. In such cases, the null hypothesis is rejected in favor of the alternative hypothesis, suggesting that at least one group mean differs from the others [59]. Conversely, when the p-value is greater than 0.05, the null hypothesis cannot be rejected, implying that the observed differences between group means may not be statistically significant and could be due to random variation. In this study, a 95% confidence level of one-way ANOVA was applied to evaluate the performance differences based on F1-score and AUC. The results are presented in Table 14 for F1-score and AUC. Each table includes the degrees of freedom (Df), sum of squares (Sum Sq), mean sum of squares (Mean Sq), F-statistic (F value), and the p-value (Pr( F)).

**Table 14:** One-way ANOVA results evaluating classifier performance at the 95% confidence level.

Dataset	Metric	Source	Df	Sum Sq	Mean Sq	F value	Pr(>F)
ECC-2013 (SMOTE)	F1-score	C(Classifier)	4.00e+00	4.29e-04	1.07e-04	1.19e+01	1.09e-06
		Residual	4.50e+01	4.05e-04	9.00e-06	—	—
	AUC	C(Classifier)	4.00e+00	2.46e-06	6.14e-07	9.92e+00	7.65e-06
		Residual	4.50e+01	2.79e-06	6.19e-08	—	—
ECC-2013 (ADASYN)	F1-score	C(Classifier)	4.00e+00	1.48e-03	3.71e-04	3.13e+01	1.76e-12
		Residual	4.50e+01	5.34e-04	1.19e-05	—	—
	AUC	C(Classifier)	4.00e+00	1.19e-05	2.98e-06	3.28e+01	8.31e-13
		Residual	4.50e+01	4.10e-06	9.11e-08	—	—
ECC-2013 (SMOTE-ENN)	F1-score	C(Classifier)	4.00e+00	1.84e-04	4.61e-05	1.05e+03	8.96e-44
		Residual	4.50e+01	1.98e-06	4.40e-08	—	—
	AUC	C(Classifier)	4.00e+00	1.81e-07	4.52e-08	1.24e+01	6.90e-07
		Residual	4.50e+01	1.64e-07	3.64e-09	—	—
ECC-2023 (SMOTE)	F1-score	C(Classifier)	4.00e+00	5.01e-03	1.25e-03	1.69e+04	8.04e-71
		Residual	4.50e+01	3.34e-06	7.42e-08	—	—
	AUC	C(Classifier)	4.00e+00	4.38e-05	1.10e-05	5.12e+03	3.44e-59
		Residual	4.50e+01	9.62e-08	2.14e-09	—	—
Australia Dataset	F1-score	C(Classifier)	4	0.0066	0.0017	0.5324	0.7125
		Residual	45	0.1397	0.0031	—	—
	AUC	C(Classifier)	4	0.0049	0.0012	0.6513	0.6290
		Residual	45	0.0851	0.0019	—	—
German Dataset	F1-score	C(Classifier)	4	0.0035	0.0009	0.0524	0.9947
		Residual	45	0.7465	0.0166	—	—
	AUC	C(Classifier)	4	0.0037	0.0009	0.0754	0.9893
		Residual	45	0.5558	0.0124	—	—
Abstract Dataset	F1-score	C(Classifier)	4	0.0005	0.0001	3.5801	0.0128
		Residual	45	0.0016	0.0000	—	—
	AUC	C(Classifier)	4	0.0000	0.0000	2.6931	0.0427
		Residual	45	0.0001	0.0000	—	—

In the one-way ANOVA results Table 14, the F1-score and AUC metrics were evaluated for various datasets. For most datasets, such as ECC-2013 (SMOTE), ECC-2013 (ADASYN), and ECC-2023 (SMOTE), the p-values are significantly smaller than 0.05 (ranging from 1.09e-06 to 9.86e-44), indicating that the null hypothesis is rejected. This suggests that the classifier performance metrics, specifically the F1-score and AUC, significantly differ across the different classification methods. For instance, in the ECC-2013 (SMOTE) dataset, the F1-score and AUC yielded p-values of 1.09e-06 and 7.65e-06, respectively, demonstrating strong statistical significance.

Conversely, in the Australia Dataset, the p-values for both F1-score and AUC are 0.5324 and 0.7125, respectively, which are greater than 0.05, indicating no statistically significant difference between the classifiers. This suggests that the observed differences between the classifier performance metrics in the Australia dataset may be due to random variation, rather than any inherent superiority of one classifier over another. Similarly, for the German Dataset, while the p-values for both F1-score and AUC are small, they are still higher than those found in other datasets (with p-values ranging from 0.0754 to 0.9893), which further supports the claim that performance differences are dataset-dependent. Overall, the results suggest that SMOTE-based methods and ADASYN consistently lead to statistically significant performance improvements in F1-score and AUC across multiple datasets, while performance differences in some datasets, such as the Australia dataset, were not significant.

In Table 15, we compared the existing models with our suggested method for all five datasets based on the accuracy, F1 score, and AUC. The F1 score gives equal weight to both precision and recall, which are measured by calculating the harmonic mean of both precision and recall. Since using only accuracy to determine a model’s correctness is not optimal, we have also utilized the F1 score and AUC.

**Table 15:** Performance comparison with other credit card fraud detection dataset.

Dataset	Ref. Paper	Approach	Performance		
			Accuracy(%)	F1 score(%)	AUC (%)
European Cardholders 2013	[65]	Ensemble voting	99.95	86.29*	-
	[50]	RF	99.96	88.39*	-
	[66]	PSO-RF	99.92	82.63	-
	[67]	RF	99.95	85.05	94.29
	[13]	Isolation Forest (IF)	99.81	54.00	-
	[1]	CNN+SGD	97.00	97.00	-	-
	[68]	Multiple Classifier	99.90	-	-
	[69]	Majority vote (Naïve Bayes + NN)	99.94	-	-
	[70]	DeepBalance	90.61	-	97.76
	[15]	ANN	99.92	78.59	-
	[71]	GAN	99.4	87.4	-
	[26]	ESMOTE-GAN RF	-	92.34	92.9
	**Our Approach**	**ET (ADASYN)**	**99.97**	**89.95**	**98.72**
European Cardholders 2023	[72]	RF	96.07	95.94	99.00
	[14]	CART + PSO	99.97	-	100
	[73]	LR (Logistic Regression)	96.00	96.00	-
	[74]	LR	99.89	-	-
	**Our Approach**	**ET**	**99.99**	**99.99**	**100.00**
Australian Dataset	[75]	Set-valued	85.56	-	-
	[25]	Extreme learning machine (ELM) and AEnet-based FS	73.5	-	88.2
	[74]	RF (KNIME)	87.50	-	-	-
	[76]	APSO-XGBoost	88.20	87.43	-
	[77]	Local rule extraction and global rule extraction	-	84.85	-
	[18]	XGBoost-TPE	87.92	-	65.71
	**Our Approach**	**AdaBoost**	**89.86**	**86.27**	**90.17**
German Dataset	[76]	APSO-XGBoost	77.48	77.96	-
	[13]	LOF (Random under-sampling)	70.60	41.00	-
	[78]	Artificial bee colony-based SVM	84.00	-	-
	[79]	Bolasso-based feature selection	84.00	-	71.3
	[80]	Ensemble	74.00	81.69	75.90
	[81]	Deep forest	81.20	-
	[18]	XGBoost-TPE	77.34	-	30.62
	[82]	KNN	75.8	-	75.89
	**Our Approach**	**Ensemble vote**	**84.00**	**89.04**	**78.30**
Abstract Dataset	[83]	Logical Graph of Behavior Profile- Enhanced Neural Network	93.10	92.53	-
	[84]	CNN	97.39	35.70	98.00
	[33]	MLP	-	81.1	85
	[33]	RUSBoost	-	40.2	96.00
	**Our Approach**	**Ensemble Vote**	**98.05**	**94.44**	**98.44**

For the European Cardholders 2013 dataset, our proposed method using (ADASYN) ET achieved an outstanding accuracy of 99.97%, an F1 score of 89.95%, and an AUC of 98.72%, marking the highest performance among the compared methods. This result highlights our model’s effectiveness in managing unbalanced class distributions. In contrast, previous techniques, including various ensemble and machine learning methods, failed to reach this level of accuracy and precision, indicating that our approach is more suited for older, complex data distributions.

Moving to the European Cardholders 2023 Dataset, we maintained the use of Extra Trees (ET) and achieved an impressive accuracy of 99.99%, further enhancing the F1 Score and AUC. This performance demonstrates the scalability and adaptability of our approach to evolving fraud detection patterns, as other models tested on this dataset did not match our accuracy, F1 scores, and AUC.

In the Australian Dataset, our implementation of AdaBoost resulted in 89.86% accuracy, an F1 Score of 86.27% and 90.17% AUC, outperforming alternative models such as ELM, XGBoost-TPE, and APSO-XGBoost. This performance underscores the efficacy of our method across diverse environments and dataset structures, while competing methods struggled to achieve high F1 scores, indicating a lack of precision necessary for effective fraud detection in this context.

For the German dataset, we employed an ensemble majority voting method, achieving 84% accuracy, 89.04% F1 score, and 78.30% AUC. This significantly outperformed models like APSO-XGBoost, LOF, and Gradient Boosted Decision Trees, underscoring the power of ensemble methods in yielding reliable results on complex, unbalanced datasets. Competing methods faced challenges in handling the intricacies and overlapping classes of the German dataset, further reinforcing the strength of our ensemble approach.

Lastly, in the Abstract Dataset, our ensemble voting method attained an accuracy of 98.05%, a 98.44% AUC, and an F1 Score of 94.44%, showcasing our method’s effectiveness even in generalized datasets with unique fraud detection challenges. Other competing methods, including CNN, Multilayer Perceptron (MLP), and RUSBoost did not achieve comparable results, suggesting that our ensemble approach provides a more reliable framework for fraud detection. This reinforces the effectiveness of our framework as a more reliable and balanced solution for fraud detection.

As such, across all five datasets, our proposed method consistently achieved the highest or near-highest accuracy, F1 scores, and AUC, proving its effectiveness in managing unbalanced class distributions and overlapping samples. The results indicate that our model not only adapts to various types of credit card fraud datasets but also provides a robust solution for diverse fraud detection scenarios.

### 6.2 Interpreting fraud detection models with SHAP

In the domain of credit card fraud detection, the interpretability of machine learning models is indispensable, fostering trust, transparency, and accountability among stakeholders. To elucidate the decision-making process of our predictive models, we employed SHAP (SHapley Additive exPlanations), a cutting-edge framework grounded in game theory [9, 43, 85]. SHAP calculates Shapley values to provide a consistent and theoretically sound measure of feature importance, quantifying the contribution of each feature to model predictions. This approach enables a deep understanding of how individual features influence the classification of transactions as fraudulent or legitimate. Through SHAP’s visualizations, such as summary plots, the distribution and magnitude of feature importance are revealed, highlighting the features that most significantly impact fraud detection. This interpretability equips financial institutions with actionable information, allowing them to understand the rationale behind the predictions of the model, improve decision-making processes, and reinforce the reliability of fraud detection systems.

**Fig 19:**
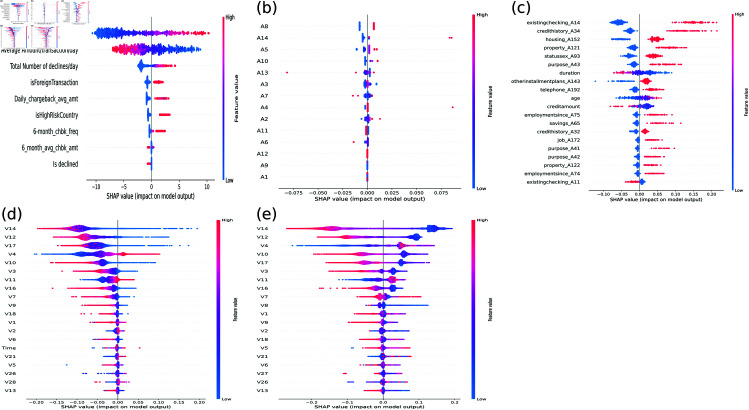
SHAP summary plots for feature importance across datasets (a) Abstract(XGBC), (b) Australia(AdaBoost), (c) German(RF), (d) ECC-2013(RF), and (e) ECC-2023(RF).

Fig 19 showcases SHAP-based feature importance analysis across five diverse datasets: European Cardholders 2013, European Cardholders 2023, the German dataset, the Australian dataset, and the Abstract dataset. Prominent features, including transaction type, age, transaction amount, and time, emerge as the most influential variables, consistently demonstrating high SHAP values. These findings affirm the critical role of these features in guiding the model’s predictions, underscoring SHAP’s efficacy in bridging the gap between model complexity and interoperability. By leveraging SHAP, we provide a transparent and comprehensive understanding of model behavior, facilitating the refinement of feature selection processes to prioritize the most impactful variables. This interpretative framework not only advances the reliability of fraud detection systems but also sets a robust foundation for developing more transparent and trustworthy AI-driven solutions.

## 7 Conclusion and future work

Detecting fraudulent credit card transactions continues to pose a significant challenge due to the severe class imbalance inherent in real-world datasets. This study introduced a novel hybrid feature selection framework combining Pearson Correlation, Information Gain (IG), and Random Forest Importance (RFI) to achieve optimal feature selection. The framework integrated state-of-the-art machine learning models, including Random Forest (RF), XGBoost (XGBC), Extra Trees (ET), CatBoost, AdaBoost, and an ensemble voting mechanism, resulting in enhanced detection accuracy. The proposed method outperformed existing baseline classifiers and approaches in recent literature, demonstrating superior performance across five diverse datasets. This robustness and adaptability underscore its potential for practical application in real-world credit card fraud detection systems.

Future research could expand this work by exploring advanced deep learning models, such as Convolutional Neural Networks (CNNs), Recurrent Neural Networks (RNNs), and Transformer-based architectures, to further improve detection capabilities. Techniques such as autoencoders for anomaly detection and Generative Adversarial Networks (GANs) for addressing data imbalance could also enhance the framework. Additionally, incorporating Federated Learning could enable privacy-preserving collaborative training across institutions, fostering globally robust fraud detection systems. Finally, leveraging real-time data streams and large-scale, contemporary datasets will allow the models to remain scalable and adaptable to the ever-evolving nature of financial fraud.
